# Risk assessment for non-carcinogenic effect posed by sulfates in water on the health of residents around The Sumpur River, West Sumatra-Indonesia

**DOI:** 10.1016/j.toxrep.2025.101921

**Published:** 2025-01-22

**Authors:** Sukarjo, Rahmah Dewi Yustika, Cicik Oktasari Handayani, Triyani Dewi, Yustiawati, Delvi Yanti, Ai Dariah

**Affiliations:** a Research Center for Horticulture, National Research and Innovation Agency Republic of Indonesia (BRIN), Jalan Raya Jakarta-Bogor Km. 46, Bogor 16911, Indonesia; b Research Center for Estate Crops, National Research and Innovation Agency Republic of Indonesia (BRIN), Jalan Raya Jakarta-Bogor Km. 46, Bogor 16911, Indonesia; c Research Center for Limnology and Water Resources, National Research and Innovation Agency Republic of Indonesia (BRIN), Jalan Raya Jakarta-Bogor Km. 46, Bogor 16911, Indonesia; d Department of Agriculture and Biosystem Technology, Universitas Andalas, Limau Manis, Padang 25166, Indonesia

**Keywords:** Human health, Risk assessment, Sulfate pollution, Water quality

## Abstract

The extensive agricultural activity contributes to runoff and plays a significant role in elevated sulfate concentrations in many global water bodies. In tropical regions, sulfate pollution and its associated health hazards have intensified, emerging as an international concern. However, these issues are often overlooked despite their potential impact on water and citizen safety. Present study intends to assess the risks posed by sulfate contamination to human health, given its critical implications for water quality in the area. The assessment was conducted through observations in seven water sampling stations established along the Sumpur River and its estuary in Lake Singkarak. The analysis of the collected samples reveals that sulfate concentrations at all locations remain within permissible limits, confirming the water's suitability for consumption. The Sulfate Pollution Index (SPI) values at all sampling locations are below 1, classifying them as unpolluted with respect to sulfate content. Additionally, the Hazard Index (HI) values at all locations were below 1, indicating no significant non-carcinogenic health risks to the public. However, location S5 recorded the highest average HI value, nearing 1 (0.95). One of sampling observations at S5, located near rice fields and settlement areas along the riverbanks, showed a value exceeding 1, which requires attention. Sustainable management of agricultural is crucial for mitigating potential health and dangers sulfate contamination and ensuring the safety of water for consumption in this region. Mitigating sulfate pollution from agriculture and residential areas requires a combination of technology, education, and regulatory enforcement. This approach should actively involve the community to create a healthier and more sustainable environment.

## 1 Introduction

Sulfate is a critical factor for both ecosystems and humans [1]. Sulfates in river water, particularly in regions with extensive agricultural activities like fertilizer and pesticide application, have become an increasing issue owing to their potential impact on the safety of people and water quality [2], [3], [4], [5]. These substances, which naturally occur in the environment, often find their way into water sources through human activities like agricultural runoff and improper household waste disposal [6], [7], [8]. While moderate sulfate levels are usually harmless, excessive concentrations can present serious health risks [9] and harmful effects on aquatic life in freshwater [10], especially in tropical areas where agriculture is intensive, and water management systems are insufficient.

Recent research has brought attention to the growing problem of sulfate contamination in aquatic ecosystems, exacerbated by industrialization [11], mining activities and rapid urbanization [12]. Studies indicate that sulfate pollution can impact processes such as carbonate weathering [13], [14], erosion [15], and the dynamics of the global carbon cycle [16]. Additionally, advancements in the use of stable isotopes have offered essential information regarding groundwater formation mechanism, sources of contamination [17], [18], [19], and pathways of sulfate pollution, enhancing our understanding of its environmental implications [6], [8].

In tropical regions such as West Sumatra, Indonesia, the Sumpur River serves as a crucial water source for local communities, providing water for drinking, agriculture, and daily household needs [20]. However, increasing sulfate levels, primarily from agricultural runoff, home sewage, industrial discharge, and systemic management deficiencies have raised serious concerns about the river's safety for human consumption [21], [22]. While awareness of water pollution is growing, the health risks associated with sulfate contamination remain underreported and require greater attention and action [23], [24].

Recent studies have underscored the critical role of addressing surface water pollution in preserving and sustaining life on Earth. This pollution occurs from many different sources classed as point and non-point sources. Points of origin refer to distinct, recognizable sources of contamination, such as pollution from factories [25], waste disposal facilities [26], and dumps [27], [28], which release contaminants straight to lakes and rivers via lines or channels, making them simple to identify and manage. Point source pollution often leads to acute contamination events, affecting local ecosystems and human health directly [29], [30]. In contrast, non-point pollutants come from extensive activity across huge regions, providing issues owing to their dispersed character and many paths of pollution, including rainwater from farms, residential stormwater, and air accumulation [31]. Temporal changes due to the impact of weather also contribute to this difficulty [32]. A pivotal study by Singh et al. [31] emphasized the urgent need to develop and implement innovative technologies for detecting and quantifying a wide range of environmental pollutants, including sulfates.

This study aims to investigate the non-carcinogenic health effects related to sulfate concentrations in the Sumpur River. By establishing multiple water monitoring stations along the river and its estuary at Lake Singkarak, the research seeks to offer a thorough investigation of sulfate levels and their potential health impacts on the local population. The outcomes of this study will enhance understanding of the sources and risks of sulfate pollution, ultimately giving actionable suggestions for environmentally friendly water use and improved community health safety in the region.

## 2 Materials and methods

### 2.1 Description of the study area

**Fig. 1:**
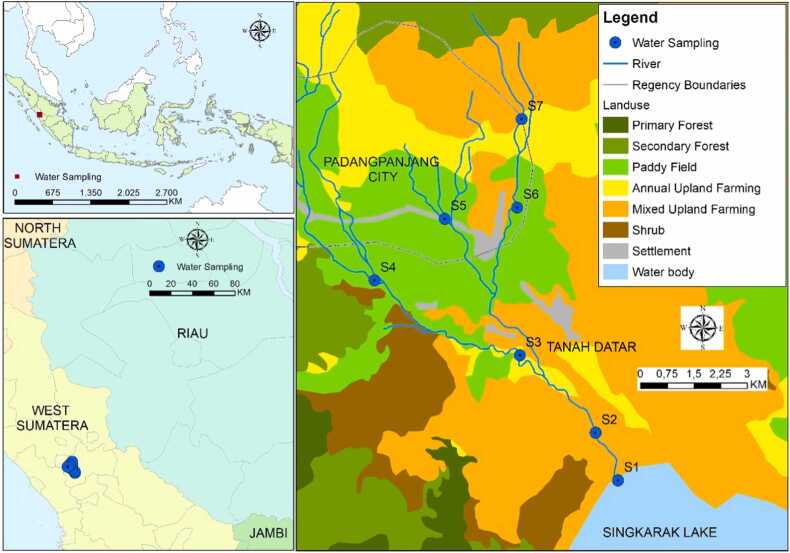
The research location, sampling points, and land use in the Sumpur Watershed.

The study was carried out in the Sumpur Watershed, located in West Sumatra, Indonesia, with its outlet flowing into Lake Singkarak (Fig. 1). Covering an area of 142.27 km², the Sumpur Watershed features diverse land uses: primary forest (3.05 %), secondary forest (14.27 %), paddy fields (22.21 %), annual upland farming (8.53 %), mixed upland farming (43.57 %), shrubland (5.36 %), and settlements (3.02 %).

### 2.2 Sampling and analysis of the sample

The collection of water was undertaken at seven locations: six points within the Sumpur Watershed (S2–S7) and one point in Lake Singkarak (S1, Fig. 1). The selected sampling points represent different river sections, including the upstream, midstream, and downstream areas and the lake near its outlet. Each point reflects the impact of surrounding land use on water quality. Point S1 represents lake land use, while point S2 reflects the effects of mixed upland farming. A combination of annual upland farming, paddy fields, and mixed upland farming influences point S3. Point S4 is primarily affected by paddy fields, whereas paddy fields and nearby settlements influence Point S5. Point S6 shows the combined impact of paddy fields and mixed upland farming, and Point S7 is influenced by annual upland farming and mixed upland farming. This study chooses these points to capture the diverse impacts of land-use practices on water quality. The sampling process adhered to the standard guidelines outlined in SNI 6989.57:2008 Section 57, which specifies methods for collecting surface water samples. River water samples were collected using a simple water sampling instrument equipped with ballast.

Water specimens were obtained in May, July, and September 2024. The samples were stored in 2-liter polyethylene bottles and kept in a cold box during transportation to ensure preservation until analysis at the laboratory. Sulfate concentrations were measured following the SNI 6989.20:2019 method at the Laboratory of the Center for Standardization and Industrial Services in Padang (Balai Standardisasi dan Pelayanan Jasa Industri – Padang).

### 2.3 Quality assurance

Sulfate assays were performed promptly after collection to avoid any metabolic transformation of the compounds. Before analysis, the materials were kept at 4°C to preserve their integrity. Quality assurance and quality control of the sulfate data, obtained through UV spectrophotometry, were ensured using a distilled water control sample. The precision of the data was further validated by reanalyzing the speciments and comparing them to the control sample.

### 2.4 Sulfate pollution index (SPI)

Sulfates primarily enter river systems via farming operations, chemical fertilizers, aquaculture, household waste, sewage, surface runoff, and floodwater. The Sulfate Pollution Index (SPI) is a dependable tool for evaluating nutrient loads and determining pollution levels in surface waters, especially in urban river environments. The SPI is calculated using the following modified equation [33]:

(1) $$\mathrm{SPI}=\;\frac{C_{s}}{{\mathrm{MAC}}_{s}}$$

where C_s_ represents the average content of sulfate in samples, and MAC_s_ indicate the maximum allowable concentration for sulfate (300 mg/L) [34].

Water quality classification depending on the SPI values is:

• SPI < 1: No pollution
• 1 < SPI ≤ 3: Moderate pollution
• < SPI ≤ 6: Significant pollution
• SPI > 6: Very high pollution

### 2.5 Human health risk assessment

The assessment of human health risks linked to two potential exposure pathways (drinking and dermal contact) of sulfate in water resources can be performed through exposure assessment [35], [36], [37]. This study utilized the Baynes [38] risk assessment framework to evaluate the non-carcinogenic risks associated with sulfate in the surface waters of the study area [7]:

(2) $$\mathrm{HI}={\mathrm{HQ}}_{\mathrm{oral}}+{\mathrm{HQ}}_{\mathrm{dermal}}=\;\frac{{\mathrm{CDI}}_{\mathrm{oral}}}{{\mathrm{RFD}}_{\mathrm{oral}}}+\frac{{\mathrm{CDI}}_{\mathrm{dermal}}}{{\mathrm{RFD}}_{\mathrm{dermal}}}$$

In Eq. 2, HI is denoted as hazard index, HQ refers to hazard quotient, CDI indicates the mean chronic daily intake (mg/kg.day), and RFD is the reference dose (mg/kg.day). RFD assesses the potential danger of sulfate in water consumption, which is 7.27 mg/kg.day, but the value for dermal exposure is half of that [38]. The reference dosage of SO_4_^2 −^ was determined as follows [39]:

(3) $$\mathrm{RFD}=\frac{\mathrm{TLV}}{\mathrm{UF}}$$

where in Eq. 3, TLV denotes the standard level of SO_4_^2-^ in water for consumption (400 mg/L) [40], UF represents a measure of security ranging from 10 to 100 (with 55 chosen as a mean for computation), and the computed RFD for SO_4_^2-^ is 7.27 mg/kg.day. Additionally, CDI was computed using the calculation method provided below [41]:

(4) $${\mathrm{CDI}}_{\mathrm{oral}}=\frac{C_{s}\;x\mathrm{IR}x\mathrm{EF}x\mathrm{ED}}{\mathrm{BW}x\mathrm{AT}}$$

(5) $${\mathrm{CDI}}_{\mathrm{dermal}}=\frac{C_{s}\;x\mathrm{SA}x\mathrm{Kp}x\mathrm{ET}x\mathrm{EF}x\mathrm{ED}}{\mathrm{BW}x\mathrm{AT}}$$

In (4), (5), C_s_ represents for examined values of sulfate, IR is the rate of consuming water which is 2.5 L/d for adults (A) and 0.78 L/d for children (C), BW is body weight for adults (A) and children (C) i.e 65 kg and 30 kg, accordingly, Kp is the dermal penetration factor in water (0.001 cm/h for both adult and children), SA is the skin surface area (10.500 and 18.000 cm^2^ for children and adult, respectively), ET is the exposure time (0.6 h/d), EF is the exposure frequency (365 d/year), ED is the exposure duration (12 and 64 years for children and adults, respectively) [42].

### 2.6 Multivariate analysis

This methodology was employed to derive the main component (PC) from the sampling location and to assess the potential sources and fluctuations of sulfate in surface water samples. Cluster analysis was used to determine the dynamics of spatial and temporal variations of sulfate concentrations [43].

## 3 Results and discussion

### 3.1 Variabilities of sulfate concentrations

**Fig. 2:**
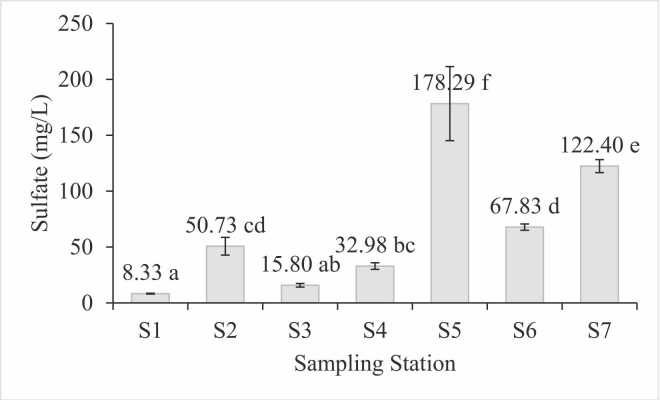
Sulfate concentration based on sampling station.

The sulfate concentrations measured at seven water sampling sites along the Sumpur River and Lake Singkarak are presented in Fig. 2. The average sulfate concentrations at the sampling locations S1, S2, S3, S4, S5, S6, and S7 were 8.33, 50.73, 15.80, 32.98, 178.29, 67.83, and 122.40 mg/L, respectively. According to Government Regulation No. 22 of 2021 [44] on Environmental Protection and Management, these concentrations are below the water quality threshold for rivers and lakes, which is set at 300 mg/L.

Site S5 exhibited the highest sulfate concentration, at 178.29 mg/L. Elevated sulfate levels can negatively impact both ecosystem and human health and may serve as a key indicator of pollution, monitoring SO_4_^2−^ sources and transport is important for water quality assessments [45]. This location is adjacent to a rice field area and settlement, which is likely a significant contributor to the elevated sulfate levels.

Sulfate is a common component of fertilizers used in rice farming to enhance soil fertility. As an essential macronutrient, sulfate supports various plant physiological functions, such as enzyme activity, protein synthesis, and chlorophyll formation [45]. Farmers often use fertilizers like ammonium sulfate (ZA), potassium sulfate (K₂SO₄), superphosphate (SP-36), and gypsum (CaSO₄) to improve soil sulfate content. Previous studies related to sulfate content in water ranged from 1.25 to 11.92 mg/L [46] and 2.83–25.00 mg/L [5], which are significantly lower than the values found in S5. Consequently, proper water management measures are essential before the surrounding population can safely utilize the water.

In addition to originating from intensive agricultural areas, sulfate pollution can also come from domestic waste, as there are settlement areas in the riparian zone flowing to point S5 (Fig. 1). Sulfate pollution from domestic waste results from the use of detergents, soaps, personal care products (such as shampoo, toothpaste, or other cleaning products containing detergents), as well as sanitation. Household waste sources of sulfates are commonly found in cleaning and personal care products, these products include additional sulfate-based synthetic compounds such as Sodium Lauryl Sulfate (SLS) and Sodium Laureth Sulfate (SLES). SLS and SLES are mostly used to create foams that enhance cleaning power [47].

In areas with poor waste disposal systems or the absence of domestic waste treatment, sulfate compounds can directly contaminate groundwater and water bodies. Tracing sulfate sources in a tropical agricultural catchment using a stable isotope Bayesian mixing model [45] revealed that domestic waste is the most dominant sulfate pollutant, contributing approximately 47 % of sulfate in groundwater and 56 % in surface water. Sulfate fertilizer (∼33 %) is the second most important source after domestic waste for groundwater, while detergents (∼23 %) are the second most significant source for surface water.

The water sampling point with the lowest sulfate concentration was S1 in Lake Singkarak, with an average value of 8.33 mg/L. This low concentration is likely due to dilution from surface runoff, the expansive volume of the lake, and sedimentation processes occurring in downstream areas [48], [49]. Additionally, natural and chemical processes during downstream transport may contribute to reducing sulfate levels [50]. Furthermore, the area between S5 and S1 passes through regions dominated by annual and mixed agricultural activities, which generally contribute less sulfate compared to rice fields. This reduced input may also help explain the lower sulfate concentrations observed at S1.

### 3.2 The sulfate pollution index (SPI)

The results of the SPI analysis were used to assess the sulfate pollution levels in the Sumpur River and Lake Singkarak (Table 1). The average SPI values across the seven water sampling locations ranged from 0.03 to 0.59. Since all SPI values are below 1, the water at these locations is classified as unpolluted in terms of sulfate content. This indicates that the sulfate levels in the Sumpur River and Lake Singkarak remain within tolerable limits.

**Table 1:** Average SPI in surface water of Sumpur River and Lake Singkarak.

Stations	Mean	SD	Min	Max	Pollution state
S1	0.03	0.00	0.03	0.03	No Pollution
S2	0.17	0.03	0.14	0.19	No Pollution
S3	0.05	0.01	0.05	0.06	No Pollution
S4	0.11	0.01	0.10	0.12	No Pollution
S5	0.59	0.11	0.51	0.72	No Pollution
S6	0.23	0.01	0.22	0.23	No Pollution
S7	0.41	0.02	0.39	0.43	No Pollution

The SPI analysis supports the findings from the sulfate concentration measurements. Sampling location S5 recorded the highest SPI value (0.59), aligning with its elevated sulfate concentration, while location S1 had the lowest SPI value (0.03), consistent with its low sulfate levels. The SPI values revealed that anthropogenic sulfate pollution is most prevalent in the S5 station, characterized by agriculture and settlement. These results confirm the overall water quality status and provide a reliable metric for evaluating sulfate pollution in the region.

### 3.3 Health risk assessment

Sulfate concentrations in the Sumpur River and Lake Singkarak were analyzed to assess the potential health risks to individuals (both children and adults) exposed to the water through two pathways: ingestion and dermal absorption. The evaluation of non-carcinogenic and carcinogenic risks was conducted by calculating the CDI values. The average CDI values for adults and children are presented in Table 2.

**Table 2:** Average CDI of sulfate for surface water in the Sumpur River and Lake Singkarak.

Water station	Age category	Ingestion		Dermal	
		Mean	SD	Mean	SD
S1	Adult	**0.32**	0.01	1.38E−3	0.06E−3
	Children	0.22	0.01	1.75E−3	0.08E−3
S2	Adult	1.95	0.31	8.43E−3	1.32E−3
	Children	1.32	0.21	1.07E−2	1.67E−3
S3	Adult	0.61	0.06	2.63E−3	0.27E−3
	Children	0.41	0.04	3.32E−3	0.34E−3
S4	Adult	1.27	0.11	5.48E−3	0.49E−3
	Children	0.86	0.08	6.93E−3	0.62E−3
S5	Adult	**6.86**	1.27	2.96E−2	5.50E−3
	Children	4.64	0.86	3.74E−2	6.96E−3
S6	Adult	2.61	0.11	1.13E−2	0.47E−3
	Children	1.76	0.07	1.43E−2	0.59E−3
S7	Adult	4.71	0.22	2.03E−2	0.97E−3
	Children	3.18	0.15	2.57E−2	1.22E−3

For adults, the highest average CDI values via water consumption and skin absorption were recorded at location S5 (6.86 mg/kg.day and 2.96E-2 mg/kg.day, respectively), while the lowest values were observed at location S1 (0.32 mg/kg.day and 1.38E-3 mg/kg.day, respectively). Similarly, for children, the highest CDI values for ingestion and dermal absorption were at S5 (4.64 mg/kg.day and 3.74 mg/kg.day, respectively), with the lowest values at S1 (0.22 mg/kg.day and 1.75E-3 mg/kg.day, respectively).

The findings indicate that among all sampling locations, S5 had the highest average CDI values for both ingestion and dermal absorption in both adults and children, while S1 (Lake Singkarak) consistently had the lowest values. These results highlight the variation in exposure risks along the Sumpur River and Lake Singkarak, with S5 being the most critical site due to its elevated sulfate levels.

Sulfate intake through ingestion plays a dominant role in the total daily sulfate exposure, as evidenced by the fact that the average CDI value via the ingestion route accounts for over 99 % of the total daily intake. This aligns with findings from similar studies, such as those by Barats et al. [51]. Although the average daily intake values for children are lower than those for adults, children still have a higher potential for sulfate intake relative to their body weight. This increased potential is due to their physiological requirements for growth and development, as highlighted by Talpur et al. [52].

Non-carcinogenic risk is evaluated using HQ and HI values, which are calculated for ingestion and dermal absorption pathways across adult and child groups. The average HQ values across all sampling locations follow the order S5 > S7 > S6 > S2 > S4 > S3 > S1 for both ingestion and dermal absorption routes (Table 3). At location S5, the highest average HQ value for ingestion is 0.94 for adults and 0.64 for children. For dermal absorption, the highest HQ values at location S5 are 8.15E-1 for adults and 1.03E-2 for children. Notably, HQ values from ingestion in adults are nearly double those in children, whereas for dermal absorption, children show higher HQ values compared to adults.

**Table 3:** Hazard Quotient (HQ) of sulfate for surface water in the Sumpur River and Lake Singkarak.

Water station	Age category	Ingestion		Dermal	
		Mean	SD	Mean	SD
S1	Adult	0.04	2.01E−3	0.38E−3	0.02E−3
	Children	0.03	1.36E−3	0.48E−3	0.02E−3
S2	Adult	0.27	4.20E−2	2.32E−3	0.36E−3
	Children	0.18	2.84E−2	2.93E−3	0.46E−3
S3	Adult	0.08	8.58E−3	0.72E−3	0.07E−3
	Children	0.06	5.80E−3	0.91E−3	0.09E−3
S4	Adult	0.17	1.57E−2	1.51E−3	0.14E−3
	Children	0.12	1.06E−2	1.91E−3	0.17E−3
S5	Adult	**0.94**	1.75E−1	**8.15**E−3	1.51E−3
	Children	**0.64**	1.18E−1	**1.03**E−2	1.91E−3
S6	Adult	0.36	1.49E−2	3.10E−3	0.13E−3
	Children	0.24	1.01E−2	3.92E−3	0.16E−3
S7	Adult	0.65	3.07E−2	5.59E−3	0.27E−3
	Children	0.44	2.08E−2	7.07E−3	0.34E−3

The aggregate HQ values for all water sampling locations and sulfate exposure pathways were found to be within permissible limits (HQ < 1). This study indicates that individuals using water from the Sumpur River and Lake Singkarak are not at significant health risk from sulfate exposure. However, a point of concern is the HQ value for adults via the ingestion route at location S5, which is notably close to the threshold of 1, with a value of 0.94. This highlights the need for proactive measures to control water consumption and reduce sulfate concentrations at location S5 to prevent potential health risks.

**Fig. 3:**
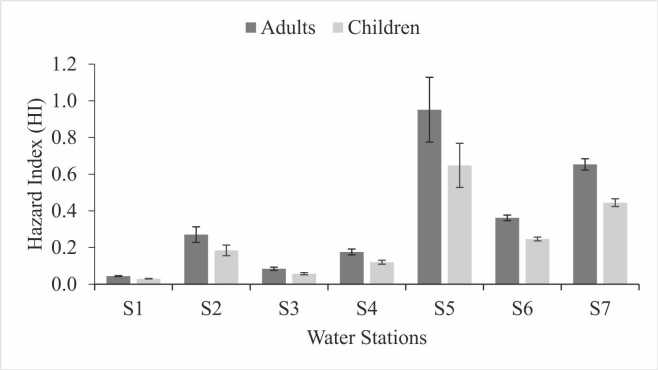
Hazard index (HI) through ingestion and dermal exposure route from different station surface water sources in the Sumpur River and Lake Singkarak.

The HI values for all sulfate exposure pathways (ingestion and dermal absorption) were evaluated based on the HQ values for both adults and children. Across the seven water sampling locations, the HI values ranked in descending order as S5 > S7 > S6 > S2 > S4 > S3 > S1 (Fig. 3). The average HI values at all locations were below one, indicating no significant non-carcinogenic health risks to the public. However, location S5 showed the highest HI values, with an average of 0.95 for adults and 0.65 for children.

It is important to note that one of the water samples from site S5 has an HI value greater than 1. This aligns with the HQ analysis at S5, emphasizing the need for caution by the local community when using water from this area. If this situation is not promptly addressed, it could pose non-carcinogenic health risks to the surrounding population, because consumption of sulfate in high concentrations over a long period of time can cause various non-carcinogenic diseases in children and adults such as diarrhea, dehydration, skin irritation, intestinal disorders, and cardiovascular disease [39], [53]. The intestines of infants and children tend to be more sensitive than those of adults, making them more susceptible to diarrhea due to consuming water with high sulfate content [54].

### 3.4 Multivariate analysis

**Fig. 4:**
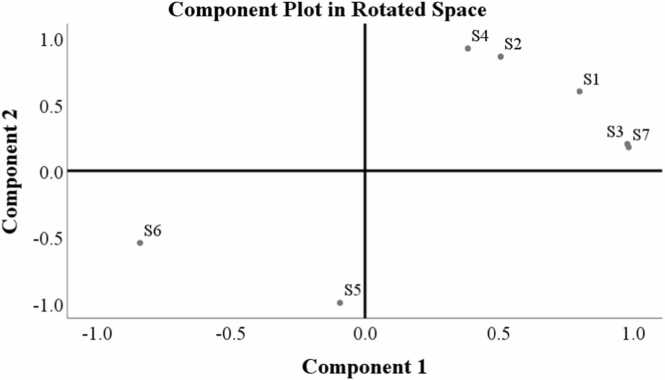
Component plot in rotated space of principal component analysis.

The PCA results shown in Fig. 4 demonstrate the distribution of sulfate properties across the seven water sampling locations (S1, S2, S3, S4, S5, S6, S7) in the rotated component space. Two distinct clusters of sampling locations are evident in the value range (> ǀ±0.5ǀ): the first group (Group 1), comprising locations S1, S3, S6, and S7 near the axial of component 1, and the second group (Group 2), including locations S2, S4, and S5 near the axial of component 2. This clustering indicates distinct sulfate patterns between the two groups, which could be attributed to variations in their sources or environmental conditions.

Sources of sulfate in water bodies can originate from a variety of natural and human-related activities, including rock weathering, volcanic activity, industrial discharge, agriculture, mining, and household waste [55]. Component Group 1, which includes locations S1, S3, S6, and S7, is located in areas characterized by mixed upland and annual upland farming land use. As a result, there is potential for additional sulfate contributions predominantly from natural sources.

In contrast, Component Group 2, encompassing locations S2, S4, and S5, is situated in areas with mixed upland and rice field farming land use. This increases the likelihood of sulfate contributions from anthropogenic activities, particularly agricultural practices.

### 3.5 Spatial and temporal variation of surface water quality

**Fig. 5:**
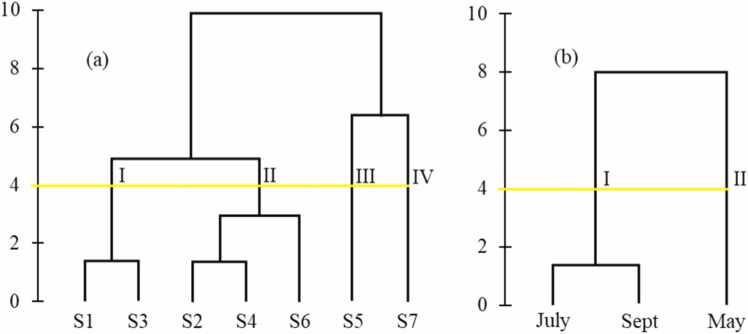
Spatial variation (a) and temporal variation (b) of surface water quality.

The cluster analysis results indicate that surface water quality at the seven monitoring sites is grouped into four clusters (Fig. 5). Clusters III and IV each represent distinct locations, corresponding to sites S5 and S7. Cluster III (S5) is the most sulfate-contaminated location compared to the other clusters (Fig. 5). Potential activities contributing to sulfate release at this site include paddy field agriculture and the settlement of household waste.

Cluster IV is formed by sampling location S7, situated upstream, where the potential sulfate sources are linked to annual and mixed upland farming. Agricultural activities, particularly paddy farming, often involve using fertilizers and pesticides that can leach into surface water, raising sulfate levels. Household waste also contributes to sulfate pollution through improper disposal and runoff [45], [56]. Studies have shown that agricultural runoff and household waste are significant sources of sulfate in water bodies [57].

Cluster I consists of monitoring locations S1 and S3. Site S1 is at the estuary of the Sumpur River and forms part of the edge of Lake Singkarak. At the same time, S3 is a monitoring site with combined land use, including paddy fields, upland farming, and mixed upland farming, located in the midstream area of the Sumpur River. Cluster II includes sampling locations S2, S4, and S6. Site S2 is downstream of the Sumpur River, with mixed upland farming as the primary land use. Site S4 is in the midstream area, where paddy fields dominate, and S6 is upstream, with a mix of paddy fields and upland farming.

Agricultural activities involving fertilizers and pesticides can lead to runoff into water bodies, increasing sulfate pollution. Mixed farming practices also contribute to nutrient runoff [58], [59]. The combination of different agricultural practices results in varied nutrient runoff, which impacts water quality differently at each location. Paddy fields and upland farming contribute to sulfate pollution through fertilizer use, while downstream sites often collect pollutants from upstream agricultural activities [56], [57].

The analysis results shown in Fig. 5 indicate that the cluster analysis from three months of monitoring formed two surface water quality clusters. Cluster I corresponds to July and September, which fall during the dry season, while Cluster II represents May, in the rainy season.

The main difference between the two clusters is the sulfate concentration, which is higher during the rainy season (Cluster II) compared to the dry season (Cluster I), as detailed in Table 4. Agricultural activities involving fertilizers and pesticides can lead to runoff into water bodies, increasing sulfate pollution. Mixed farming practices also contribute to nutrient runoff [56].

**Table 4:** Mean values of sulfate concentration in the identified clusters.

Cluster	Spatial Variation		Temporal variation	
	Member of cluster	Concentration (mg/L)	Member of cluster	Concentration(mg/L)
I	S1, S3	12.07 ± 14.22	July, Sept	66.35 ± 54.10
II	S2, S4, S6	50.52 ± 15.17	May	71.46 ± 74.22
III	S5	178.29 ± 33.12		
IV	S7	122.40 ± 5.81		

Higher sulfate concentrations during the rainy season compared to the dry season have also been observed in Chennai Lake in India, reservoirs in Ethiopia, and the surface waters of the Fen River on the eastern Chinese Loess Plateau [60], [61], [62]. The increase in sulfate concentrations in river surface water during the rainy season can be attributed to several sources.

First, increased runoff from surrounding areas can transport sulfate from natural sources, such as soil and rocks, and anthropogenic activities, including agricultural and industrial processes. Second, heavy rainfall can cause soil and rock erosion, often containing sulfate minerals. These eroded materials are then carried into rivers, elevating sulfate concentrations.

Urban areas exacerbate this increase through stormwater drainage systems that collect and direct rainfall, which may carry sulfate from various sources, such as industrial waste and sewage. Agricultural activities further increase sulfate levels, as fertilizers and other chemicals can be washed into rivers after heavy rains. Lastly, industrial waste from factories and facilities may contain sulfate, and its discharge often increases during the rainy season due to higher water flow.

The combination of these factors leads to higher sulfate concentrations in the surface waters of the Sumpur River and Lake Singkarak during the rainy season. Increased rainfall intensifies the flow of sulfate sources such as agricultural runoff, industrial waste, household waste, and other community activities [60].

### 3.6 Policy and practical implications

Mitigating sulfate pollution in areas identified as contaminated, such as the S5 site, which predominantly has agricultural practices of paddy fields, is recommended to monitor sulfate levels in groundwater, irrigation channels, and surrounding water bodies. Alongside monitoring, the implementation of targeted pollution control measures is essential. In agricultural areas, it is advised to avoid excessive fertilizer application and integrate organic fertilizers to meet crop nutrient requirements while promoting sustainable soil management [63], [64]. Site-specific nutrient management, which considers the balance between nutrient supply and crop demand, can help achieve high productivity while minimizing nutrient wastage [65]. Applying the appropriate dosage ensures plants receive sufficient nutrients without causing toxicity or creating nutrient imbalances in the soil. Over-application can result in nutrient leaching, water pollution, and increased greenhouse gas emissions, whereas under-application leads to suboptimal crop performance. Timing is equally critical; applying sulfur fertilizers during key phenological phases ensures optimal nutrient uptake [66]. Split applications can further minimize nutrient losses and enhance crop yields [67]. Additionally, proper fertilizer placement—such as positioning fertilizers near the root zone—ensures direct nutrient availability to plants, reducing wastage.

Other recommendations to minimize sulfate levels in agriculture areas include the use of slow-release fertilizers to reduce sulfate leaching into water systems [68] and the adoption of conservation techniques, such as contour farming and buffer strips, to minimize surface runoff that carries pollutants. By adopting these practices, farmers can improve productivity while promoting sustainable agricultural systems.

For residential areas, the recommended measures include: (1) construct septic tanks or closed sanitation systems to prevent direct groundwater and surface water contamination, (2) ensure waste disposal systems include effective treatment processes before discharge into the environment, (3) minimize sulfate-containing products, such as detergents, and promote environmentally friendly alternatives.

Public education initiatives should be introduced to raise awareness about the environmental and health impacts of excessive sulfate use. These programs can promote behavioral changes and foster community participation in efforts to reduce pollution. Lastly, the strict enforcement of environmental regulations is essential to ensure compliance with pollution control measures and to support long-term improvements in water quality.

## 4 Conclusion

The research results indicate that the sulfate pollution index values at several water sampling locations (S1, S2, S3, S4, S5, S6, S7) are still less than one, categorizing these areas as unpolluted or indicating that the sulfate concentrations in the Sumpur River and Lake Singkarak are within acceptable limits.

There are no non-carcinogenic health risks to public health from sulfate exposure in the Sumpur River and Lake Singkarak waters, as the HI and HQ values are less than 1 for both adults and children. However, at location S5 influenced by the agricultural practice of rice farming, the HQ and HI values are nearing 1, underscoring the need for measures to reduce water consumption rates and lower sulfate concentrations in this area.

Sulfate sources at locations S1, S3, S6, and S7 are predominantly derived from natural processes, whereas locations S2, S4, and S5 are significantly influenced by anthropogenic activities, particularly household waste and agricultural practices associated with mixed upland and rice field farming. To address this issue, further research is recommended to pinpoint the specific sources of sulfate pollution and to develop targeted mitigation strategies. This should include evaluating the effectiveness of various pollution control measures and exploring innovative technologies for sulfate removal to ensure long-term water quality and public health safety. Implementing targeted pollution control measures and avoiding excessive fertilizer application is essential for mitigating sulfate pollution in agricultural areas like the S5 site. Constructing septic tanks and promoting eco-friendly products are recommended to prevent sulfate contamination in residential areas.

## Funding

This research received grant funding from the Research Organization for Earth Sciences and Maritime, BRIN, under grant number 6/III/HK/2024.

## CRediT authorship contribution statement

**Sukarjo:** Writing – review & editing, Writing – original draft, Visualization, Validation, Methodology, Formal analysis, Conceptualization. **Rahmah Dewi Yustika:** Writing – review & editing, Writing – original draft, Supervision, Methodology, Funding acquisition, Data curation, Conceptualization. **Cicik Oktasari Handayani:** Writing – original draft, Visualization, Project administration, Methodology, Formal analysis, Data curation, Conceptualization. **Triyani Dewi:** Writing – original draft, Methodology, Formal analysis, Data curation, Conceptualization. **Yustiawati:** Writing – original draft, Validation, Methodology, Data curation. **Delvi Yanti:** Writing – original draft, Data curation. **Ai: Dariah:** Writing – review & editing, Writing – original draft, Methodology, Conceptualization.

## Declaration of Competing Interest

The authors declare that they have no known competing financial interests or personal relationships that could have appeared to influence the work reported in this paper.

## Data Availability

Data will be made available on request.
